# Home range utilization by chacma baboon (*Papio ursinus*) troops on Suikerbosrand Nature Reserve, South Africa

**DOI:** 10.1371/journal.pone.0194717

**Published:** 2018-03-29

**Authors:** Kerry Slater, Alan Barrett, Leslie R. Brown

**Affiliations:** Applied Behavioural Ecology and Ecosystem Research Unit, Department of Environmental Sciences, University of South Africa, Florida, Republic of South Africa; Sichuan University, CHINA

## Abstract

Rapid urbanization coupled with decreasing areas of natural habitat are causing baboon populations to become scattered and isolated, often resulting in increased levels of human-baboon conflict. To implement baboon-human conflict management strategies, it is essential to formulate realistic conservation policies that deal with all stakeholder concerns and ensure the conservation of viable baboon populations. A study was initiated in response to complaints of perceived excessive baboon numbers and associated lack of food resources on Suikerbosrand Nature Reserve in South Africa. Data obtained from GPS tracking collars fitted to one baboon from each of 10 identified troops were analyzed to determine home range size and utilization. The spatial representation of home ranges generated from this study will allow reserve management to identify areas of potential high and low human-baboon conflict and will contribute to the development of a formal baboon management plan to reduce human-baboon conflict on and around the reserve. Home ranges were unevenly distributed and had a mean size of 26.72 km^2^ ± 13.91 SD in the cold/dry season and 26.54 km^2^ ± 12.76 SD in the warm/wet season. Troop home ranges overlapped to some degree and five troops utilized areas outside the reserve. Although no significant relationship between troop size and home range was found, there was a positive relationship between troop size and daily distance travelled. All troops had significantly longer mean daily distances during the warm/wet season than during the cold/dry season (P ≤ 0.02).

## Introduction

Globally the rapid increase in urbanization accompanied by decreasing natural habitats are causing many animal species, including primates, to become scattered and isolated. Animal populations require a certain amount of resources to meet their daily metabolic demands. Variation in intraspecific home range sizes and distances travelled per day are influenced by both habitat diversity and seasonal changes in resource availability [1]. Primates living in habitats with low levels of food availability or relatively homogeneous habitats normally have larger home ranges than those in areas with high levels of food availability or heterogeneous habitats [2]. Furthermore, seasonal changes in resource availability are predicted to influence seasonal home range size [2]. The number of animals in a troop is an additional factor influencing home range size. Larger troops experience increased intragroup competition for resources than smaller troops, which results in larger daily distances travelled. [3, 4, 5, 6, 7, 8].

Chacma baboons (*Papio hamadryas ursinus*) are a widespread and ecologically flexible primate species distributed throughout southern Africa. They can survive in a variety of habitats, with variable environmental conditions [9] and are able to exploit diverse food supplies, suitable sleeping sites and water sources [4, 5]. Resources used by chacma baboons fluctuate both temporally [10] and spatially across seasons [11], resulting in baboons having to adjust their food intake accordingly and therefore baboons need to modify their use of resources as and when resources are available [12]. For example, plant parts such as fruit and flowers are only available for short periods at certain times of the year (depending on the plant species), and can therefore only be exploited when they are available, resulting in limited access to these items [4]. As eclectic omnivores [13, 14, 15, 16, 17, 18] baboons are known to include human food into their diet when available [19, 20, 21, 22].

Baboons are highly mobile within their home ranges and several troops living in an area can have variable home range sizes, indicating seasonal differences with regard to localized food availability, troop size and structure [12, 20, 21]. In areas where more than one troop occurs, baboons do not appear to defend territories and it is common for home ranges of neighboring troops to overlap [23, 24]. Seasonal availability of resources affects not only the distance travelled to obtain required resources but also foraging effort (i.e. time and/or energy an animal allocates to foraging to meet energy needs under changing environmental conditions) [2, 25]. Chacma baboons living in mountainous areas travel longer daily distances when food resource levels are at their lowest towards the end of winter [26]. Yellow baboons (*P*. *hamadryas cynocephalus*) in Tanzania increased both their foraging time and daily distance travelled when rainfall and daily temperatures were low, but these distances decreased with higher rainfall and temperatures [27, 28]. The degree of habitat heterogeneity or homogeneity within a troop’s home range will also influence movement patterns [2]. In homogeneous habitats where resource dispersion is uniform, baboons spend less time travelling and more effort actively foraging [4], but when resources are patchily distributed as in heterogeneous habitats, foraging effort will reflect the distance needed to travel to reach a food patch, rather than time spent actively foraging within a patch [29].

One of the consequences of baboons’ ecological flexibility is that they are able to exploit human-modified habitats [30]. Baboons that live in close proximity to human-modified landscapes typically modify their diets to include high-energy human-derived foods, which they obtain from agricultural fields, garbage disposal sites, rubbish bins, houses, and picnic or camping sites [19, 30, 31]. Increased foraging efficiency and food quality may subsequently result in an increase in both growth and reproduction [32], but can also expose baboons to new diseases [33]. During ‘raiding behaviour’ damage to infrastructure may occur (e.g. milking machines on dairy farms [19]) and in extreme instances people and domestic animals have been attacked by raiding baboons [19]. This ‘raiding’ behaviour of baboons gives rise to various levels of human–baboon conflict that often causes people to wound, maim or kill baboons [34]. If regular shooting of baboons targets large males, a troop’s composition can change to that of a female biased demography [35], which may have social, genetic dispersal and reproductive implications [36]. With a constantly increasing human population resulting in reduced and fragmented natural landscapes, increased levels of human–baboon co-existence and associated conflict are inevitable [24, 30, 34, 37, 38].

To implement baboon–human conflict management strategies, it is essential to formulate locally appropriate conservation policies that deal realistically with all stakeholder concerns, but also to ensure the conservation of viable baboon populations [39]. However, for such policies to be viable, they must be developed using scientific knowledge about the ecology of baboons. One of the key components to understanding baboon ecology is having a visual representation of their seasonal spatial use within their habitat. Once this representation is available, more detailed information such as preference-avoidance for certain plant communities, predator avoidance, and availability of resources such as food, water and sleeping sites can be identified. Having this information available, reserve management will be able to identify low and high baboon utilization areas, which can be translated into potentially low and high human-baboon conflict areas. Once these areas of potential conflict are identified, plans for additional tourist facilities on the reserve can consider this information and avoid building tourist facilities in areas on the reserve which are considered high baboon-human conflict areas. Furthermore, by quantifying areas outside the reserve that the baboons utilize, and at which times of the year these areas are likely to be utilised, mitigation methods to reduce conflict can be introduced. Until such a time that baboon habitat and land use patterns are incorporated into management plans, the management of baboons will continue to be based on reactions to sporadic crises.

We studied 12 baboon troops inhabiting the Suikerbosrand Nature Reserve (SNR), Gauteng, South Africa to investigate their spatial ecology. The SNR is made up of large tracts of indigenous vegetation interspersed with fallow lands. Areas adjacent to the reserve contain a mixture of natural vegetation, agricultural lands (livestock and crops) and urban residential areas. The reserve provides an ideal testing ground for investigating some of the above-mentioned issues as it is a formally protected area and a popular tourist facility. Furthermore, it is surrounded by varying levels of human-modified habitat.

Our study objectives were to determine the spatial utilization patterns of baboon troops residing in the Suikerbosrand Nature Reserve, by quantifying their home range sizes, daily distances travelled and areas of high versus low utilization. This information will provide management with baseline information on the movement of baboons on Suikerbosrand Nature Reserve, as well as identify further research questions that can be incorporated into future management plans.

## Materials and methods

### Study site

Suikerbosrand Nature Reserve (SNR) is a protected area of approximately 179.90 km^2^ situated about 50 km southeast of Johannesburg, Gauteng Province, South Africa (−26.4379S, 28.2193E) with an altitude ranging from 1545 to 1917 m above sea level. The reserve is dominated by the Suikerbosrand mountain range and is surrounded by various forms of development ranging from open veld to agricultural farmland and urban settlements. The geology of SNR consists of two geological systems: the Ventersdorp system (volcanic origin) which covers approximately 70% of the reserve and the Witwatersrand system (sedimentary) which covers 30% [40]. Rocky outcrops which belong to the lower Proterozoic time period (3 000 to 1 650 MYA) are common on mountain slopes and peaks. There is a vlei in the south, a dam of about 200m^2^ in the east, and several perennial rivers and permanent water sources scattered throughout the reserve. A range of landscape units ranging from plateaus, scarps, mid-slopes, foot-slopes and valley floors are also present on the reserve. Suikerbosrand falls within the summer rainfall region of South Africa, with an annual rainfall of between 650 mm and 700 mm per year. Most rain falls between October and March [18]. For the purpose of this study, two seasons were distinguished: the warm/wet season from the beginning of November to the end of April, and the cold/dry season from the beginning of May to the end of October. For the period 2000–2006, temperatures ranged from 7.5°C to 31°C during the warm/wet season and from -3°C to 33°C during the cold/dry season [18].

Vegetation units that have been identified on SNR include: Andesite Mountain Bushveld, Tsakane Clay Grassland and Gold Reef Mountain Bushveld [41]. Within these broad vegetation units, eight broad plant communities have been identified: *Stoebe vulgaris-Eragrostis plana*, *Harpochloa falx Indigofera hedyantha*, *Cussonia paniculata subsp*. *paniculata-Hermannia grandistipula*, *Acacia caffra-Ehrharta erecta var*. *natalensis*, *Acacia karroo-Panicum maximum*, *Leucosidea sericea-Setaria sphacelata var*. *sericea*, *Englerophytum magalismontanum-Aristida transvaalensis* and old agricultural lands [42].

### Data collection

#### Ethics statement

The original study that involved the capturing, immobilizing and fitting of collars onto baboons was conducted by SNR [43] with approval from the Gauteng Department of Agriculture and Rural Development’s (GDARD) internal research committee. For the purpose of this paper, permission to utilize the secondary data was granted by the Suikerbosrand Nature Reserve's Management Committee, and approved by the University of South Africa’s Animal Ethics committee (Ref no. CAES14/10/2010).

#### Study population

As part of the ongoing management of SNR a census of the baboon population was conducted during October 2006 indicating that there were 12 baboon troops with a total number of between 611 and 764 baboons on the reserve [43], with a density of between 3.39 and 4.25 baboons/km^2^. Data collected by the Gauteng Department of Agriculture and Rural development (GDARD) on the movements of the 12 troops were utilized for the purposes of this study.

Movement data were collected by fitting cell-phone telemetry collars to one female from each of the 12 identified troops. Baboons were caught using baited (with vegetables) cage traps that were set up close to known baboon sleeping sites across the reserve. When a baboon was captured, the cage was covered with a tarpaulin to reduce stress and the animal was immobilized by a departmental veterinarian. Baboon troops generally move through their home ranges as a cohesive unit with females being less likely to change troops than males [27]. With this in mind, only one female per identified troop was fitted with a tracking collar and the movement of the one collared baboon within each troop represented the movement patterns of the whole troop. Due to the limited life span of batteries, the tracking collars were set to record location co-ordinates four times during daylight hours and once at night. Co-ordinates were sent via SMS to a GSM network where they were downloaded and stored in a central database for future retrieval. If cell-phone coverage was unavailable, each collar would store up to 240 GPS co-ordinates and once the collared individuals were within the range of a cell phone mast, the stored co-ordinates were then transmitted [19]. Of the 12 collars that were initially fitted to the baboons, two malfunctioned and therefore only data downloaded from 10 tracking collars and hence 10 troops were used for analysis. The collar ID’s were used to represent each troop. For example if a female was fitted with collar AS33, then the troop to which she belonged was known as troop AS33. Details of each troop’s identification code and size [43] is presented in Table 1.

**Table 1:** Home range size (km^2^) and percentage of intra-troop overlap on Suikerbosrand Nature Reserve using 100% and 50% Minimum Convex Polygons (MCP) (n = number of GPS points used to calculate 100% MCPs).

	Range size (km^2^)								
		Seasons combined		Cold/dry season		Warm/wet season		Overlap (%) between seasons	
Troop ID	Troop size	100% MCP	50% MCP	100% MCP	50% MCP	100% MCP	50% MCP	100% MCP	50% MCP
AS33	24	17.73 (n = 1724)	2.52	17.05 (n = 828)	2.31	13.89 (n = 896)	2.01	75.26	71.26
AS35	23	21.81 (n = 1486)	4.04	21.06 (n = 658)	2.26	18.35 (n = 828)	2.58	81.61	19.93
AS36	70	60.27 (n = 1377)	17.46	57.24 (n = 647)	17.08	49.89 (n = 730)	7.42	77.20	40.34
AS39	46	25.48 (n = 1598)	10.49	22.79 (n = 699)	8.39	19.98 (n = 899)	7.63	68.12	52.67
AS41	54	18.69 (n = 1270)	3.98	13.74 (n = 496)	3.16	17.22 (n = 744)	2.36	66.29	38.73
AS42	20	42.99 (n = 1545)	7.78	25.93 (n = 782)	5.80	41.56 (n = 763)	4.61	57.41	33.73
AS43	64	22.65(n = 1636)	6.41	20.79 (n = 865)	5.98	17.80 (n = 771)	3.19	70.13	43.05
AS45	33	50.57 (n = 1501)	14.37	46.66 (n = 807)	9.13	41.30 (n = 694)	8.00	73.96	19.22
AS55	29	25.07 (n = 1713)	6.46	20.10 (n = 851)	6.22	20.10 (n = 862)	2.58	64.81	36.20
AS56	18	28.56 (n = 1557)	5.07	21.04 (n = 855)	2.42	25.34 (n = 702)	4.47	62.29	35.93

### Data analysis

For the purpose of this study 12 months of data were analysed and two seasons were distinguished. The warm/wet season was considered to be from the beginning of November to the end of April and the cold/dry season from the beginning of May to the end of October. The package QGIS desktop (vs 2.10.1-Pisa) was used to overlay the GPS co-ordinates of the collared females (troops) onto a map of the SNR and surrounding areas. We used Minimum Convex Polygons (MCP) [44], to calculate annual and seasonal home ranges at both 100% and 50% MCP levels. From these home ranges, the size and percentage of home range overlap both within and between troops for each season was calculated using Biotas™ home range software [45]. Daily distances travelled were calculated by measuring the distance between each successive GPS location recorded by the tracking collar for each day during the study period.

Wilcoxon signed rank (matched) tests were used to determine if each troop’s home range size and daily distances travelled differed between the cold/dry and warm/wet season. Wilcoxon rank sum tests (unmatched) were used to determine if there were significant differences in home range sizes and daily distances travelled between troops during the cold/dry or warm/wet season. Spearman rank correlations were used to determine if there was a seasonal relationship between troop size and home range size. To obtain an estimate of the percentage of time each troop spent inside the reserve, the percentage of each troop’s GPS co-ordinates that fell inside and outside SNR was calculated.

## Results

### Home range size

**Fig 1:**
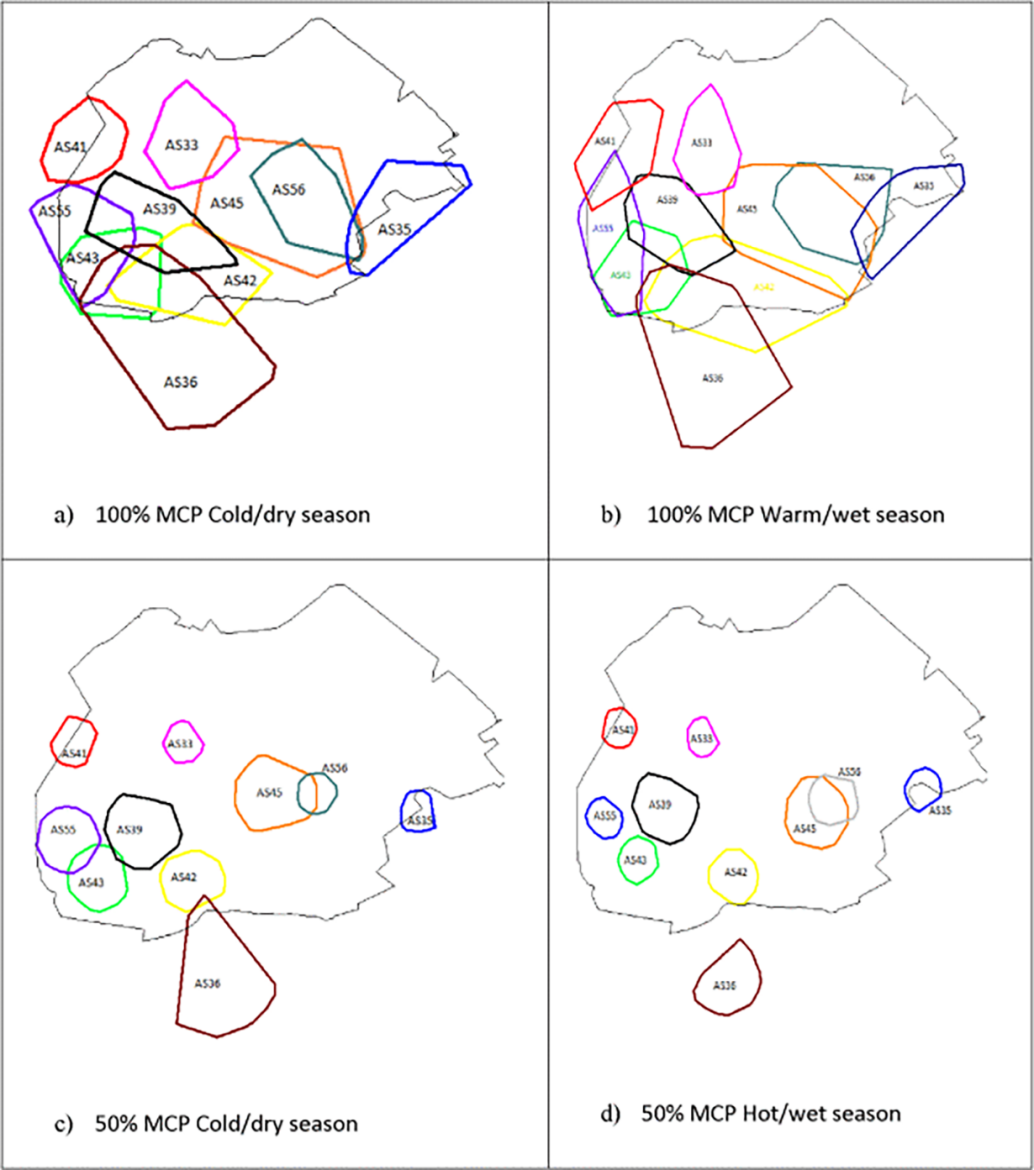
Seasonal home ranges for 10 chacma baboon troops on Suikerbosrand Nature Reserve.

The home ranges determined for the 10 baboon troops during the study period varied in terms of size and location within the study area with six of the troops utilizing areas outside the reserve to some extent (Fig 1). Total home ranges (100% MCP) ranged from 13.74 km^2^ to 57.24 km^2^ (mean: 26.72±13.91 SD) in the cold/dry season and from 13.89 km^2^ to 49.89 km^2^ (mean: 26.54±12.76 SD) in the warm/wet season (Table 1). Core home ranges (50% MCP) ranged from 2.26 km^2^ to 17.08 km^2^ (mean: 6.27±4.55 SD) in the cold/dry season and from 2.01 km^2^ to 8.00 km^2^ (mean: 4.48±2.37 SD) in the warm/wet season (Table 1). Results from Wilcoxon rank sum tests showed no significant differences in home range size between troops (annual: Z = 0.69, P = 0.49, N = 45; warm/wet: Z = 0.75, P = 0.45, N = 45; cold/dry: Z = 0.11, P = 0.92, N = 45). There were no significant differences in the home range size between the warm/wet compared to the cold/dry season for any of the troop’s home range size for either the total range (Wilcoxon signed rank test: Z = -0.46, P = 0.645, N = 10) or core range (Z = -1.89, P = 0.0588, N = 10) calculated areas. The degree of overlap between a troop’s cold/dry and warm/wet season home range ranged from 57.41% (12.39 km^2^) to 81.61% (46.53 km^2^) (mean: 69.70%±7.38 SD) for total ranges and from 19.22% (0.80 km^2^) to 71.26% (7.04 km^2^) (mean: 39.11%±15.08 SD) for core ranges (Table 1).

For total home ranges (100% MCP), all troops had overlapping home ranges with at least one other troop (Table 2), but for core ranges (50% MCP) seven troops had overlaps with at least one other troop during the cold/dry season, and two troops had overlapping core ranges with each other during the warm/wet season (Table 3). Total range overlap between troops in the warm/wet season ranged from between 0.18 and 53.7% (mean: 12.06±15.34 SD) and in the cold/dry season between 0.04 and 45.34% (mean: 11.60±12.62 SD) (Table 2). There was no significant difference in the seasonal size of home range overlap between troops that had overlapping home ranges. (Wilcoxon rank sum: Z = 0.16, P = 0.87, N = 14)

**Table 2:** The size (km^2^) and percentage of seasonal home range overlap between baboon troops on Suikerbosrand Nature Reserve based on total range (100% MCPs). Troops with no overlapping home ranges are excluded.

	Home range size of both troops combined (100% MCP)		Home range overlap (%)	
Overlapping troop ID’s	Warm/wet season	Cold/dry season	Warm/wet season	Cold/dry season
AS33 and AS39	33.75	No overlap	0.24	No overlap
AS33 and AS45	55.28	62.02	0.18	2.81
AS35 and AS45	57.55	66.02	3.99	2.33
AS35 and AS56	39.54	40.50	10.71	3.00
AS36 and AS39	69.56	77.25	0.24	3.13
AS36 and AS42	75.48	69.99	21.80	19.14
AS36 and AS43	63.51	65.44	7.55	19.50
AS36 and AS55	68.82	75.18	0.48	4.22
AS39 and AS41	36.99	No overlap	0.42	No overlap
AS39 and AS42	56.97	40.99	8.31	19.64
AS39 and AS43	33.72	39.35	53.72	10.50
AS39 and AS45	0.00	68.84	0.00	0.35
AS39 and AS55	38.31	38.99	4.87	12.41
AS41 and AS55	33.55	No overlap	10.73	No overlap
AS42 and AS43	55.37	41.15	7.82	13.86
AS42 and AS45	79.34	72.37	5.33	0.18
AS42 and AS55	0.00	46.78	0.00	0.04
AS43 and AS55	29.15	32.39	30.11	29.09
AS45 and AS56	48.18	46.93	18.59	45.34

**Table 3:** The size (km^2^) and percentage of seasonal core range overlap between baboon troops on Suikerbosrand Nature Reserve based on 50% MCPs. Troops with no overlapping home ranges are excluded.

	Core range size (km^2^) of both troops combined (50%MCP)		Core range overlap (%)	
Overlapping troop ID	Warm/wet season	Cold/dry season	Warm/wet season	Cold/dry season
AS36 and AS42	0.00	22.76	No overlap	0.53
AS39 and AS43	0.00	14.27	No overlap	0.69
AS43 and AS55	0.00	12.17	No overlap	5.79
AS45 and AS56	9.42	10.43	No overlap	32.44

### Home range size and troop size

Although we expected home range size to increase as troop size increased, Spearman rank correlations showed no significant relationship between troop size and overall (r_s_ = 0.06, N = 10, P = 0.86), warm/wet season (r_s_ = -0.07, N = 10, P = 0.86), or cold/dry season (r_s_ = 0.01, N = 10, P = 0.97) total home ranges. No significant relationship was found for troop size and core range either: overall (r_s_ = 0.47, N = 10, P = 0.17), warm/wet season (r_s_ = 0.18, N = 10, P = 0.61), or cold/dry season (r_s_ = 0.64, N = 10, P = 0.05).

### Daily distances travelled

**Fig 2:**
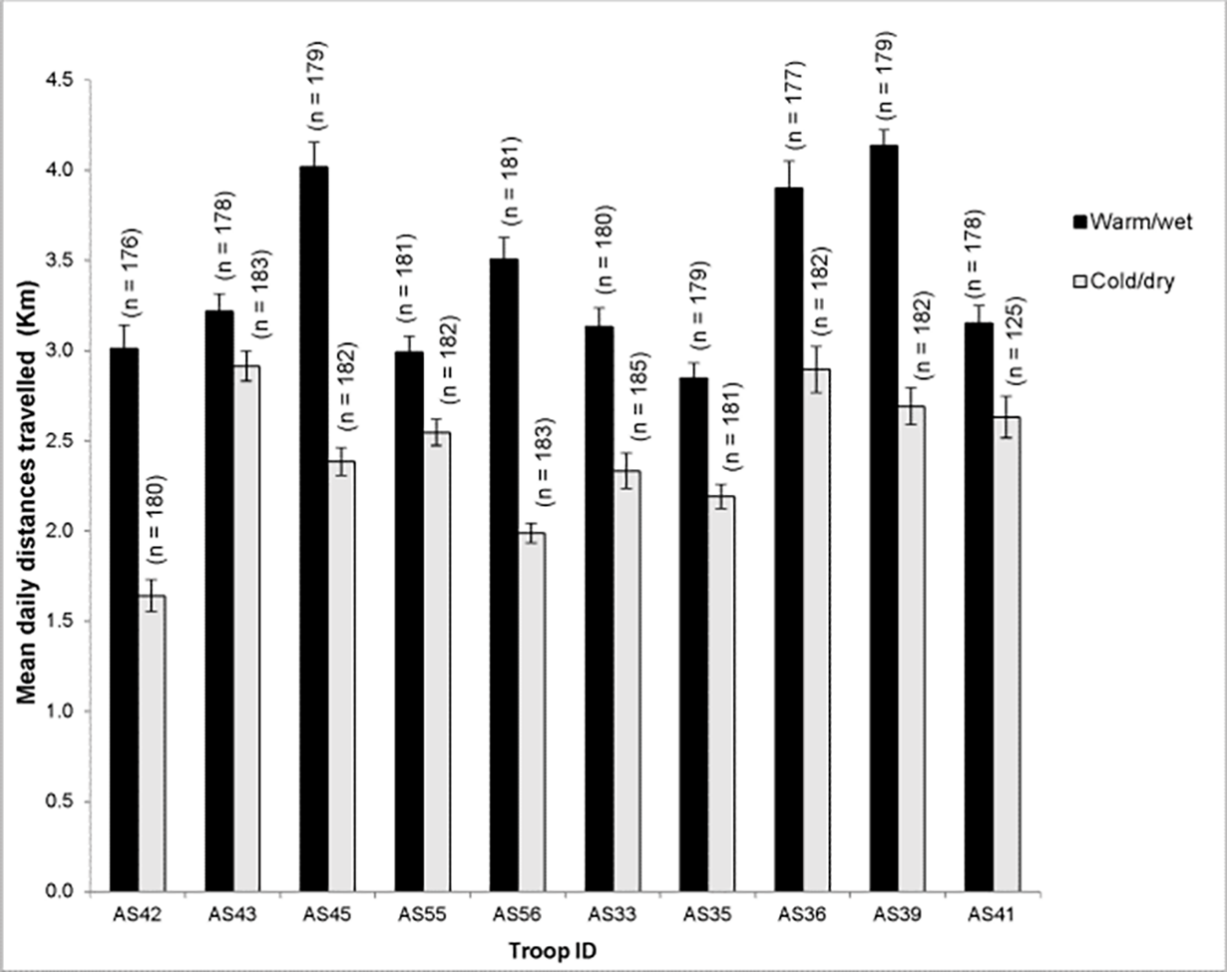
Mean ± SE daily distances travelled by baboon troops on Suikerbosrand Nature Reserve during the warm/wet and cold/dry season (n = number of days).

Mean daily distances travelled by the troops ranged from 1.64 km (±0.09 SE) to 2.91 km (±0.09 SE) during the cold/dry season and from 2.85 km (±0.08 SE) to 4.14 km (±0.09 SE) during the warm/wet season. Wilcoxon signed rank tests showed that all troops travelled significantly longer mean daily distances during the warm/wet season than during the cold/dry season (P ≤ 0.02 for all troops) (Fig 2).

We found that for daily distances travelled during the cold/dry season, 40 of the 46 (86.96%) intertroop comparisons had significantly different distances (Kruskall-Wallace ANOVA: F = 21.66, df = 9, P<0.001). For the warm/wet season we found that 41 of the 46 (89.13%) possible combinations had significantly different daily distances (F = 19.13, df = 9, P<0.001).

### Daily distances and troop size

Significant positive relationships between troop size and daily distances travelled were found for both the cold/dry (Spearman rank: r_s_ = 0.95, N = 10, P<0.001) and warm/wet season (r_s_ = 0.45, N = 10, P = 0.18).

### Ranging within and outside reserve

**Fig 3:**
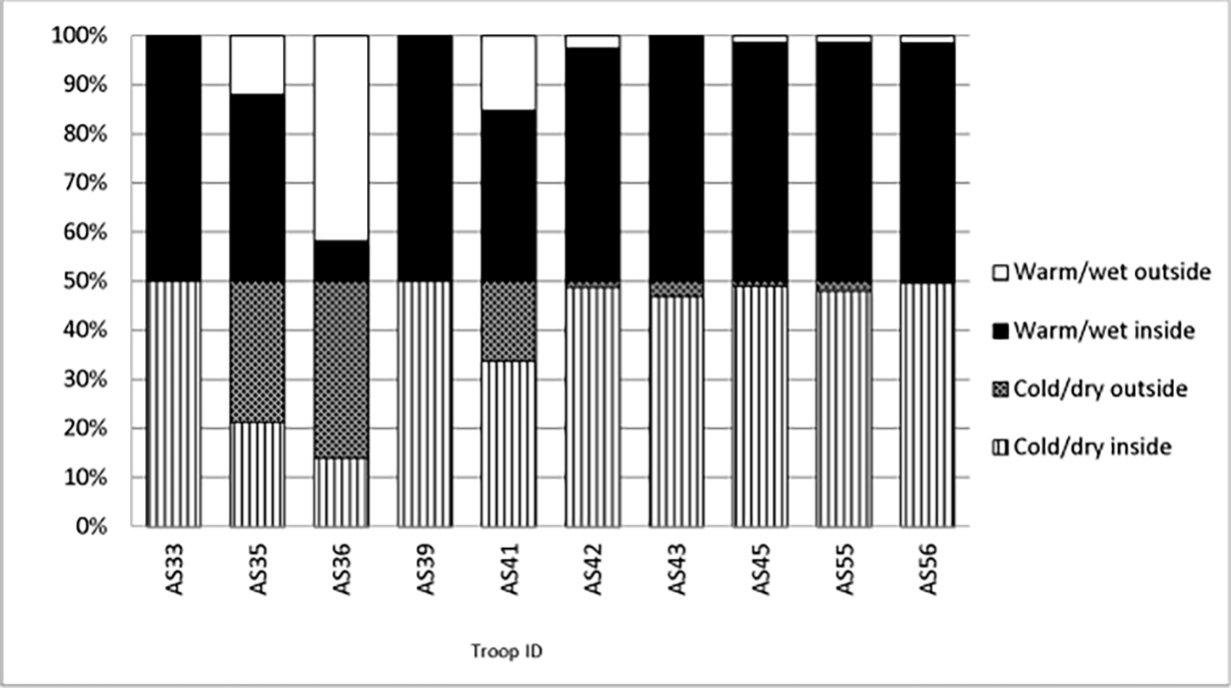
Percentage of GPS locations recorded for each troop that fell within or outside the boundary fence of Suikerbosrand Nature Reserve during both the warm/wet and cold/dry seasons.

The percentage of recorded GPS locations for each of the troops that occurred outside of the reserve varied between troops (Fig 3). Based on the percentage of a troop’s GPS points occurring outside of SNR, the tendency for a troop to leave SNR was regarded as low (<10%), medium (11–50%) or high (>50%). Based on this categorization, the following troops had a low tendency to leave the reserve during the cold/dry season: AS42, AS43, AS45, AS55, AS56 and during the warm/wet season: AS42, AS45, AS55, AS56. Troop AS35 had 24% of its GPS points recorded outside the reserve during the warm/wet season. For troop AS41, 33% of its GPS points were recorded outside the reserve during the cold/dry season and 31% during the warm/wet season. Troops that had a high tendency to move out of the reserve during the cold/dry season were AS35 and AS36 but only AS36 was recorded leaving the reserve frequently during the hot/wet season. Troops AS33 and AS39 were never recorded leaving the reserve.

Although there were positive correlations between troop size and percentage of GPS points recorded outside of the reserve, correlations were not significant for either the cold/dry (Spearmans: r_s_ = 0.43, N = 10, P = 0.218) or hot/wet (r_s_ = 0.09, N = 10, P = 0.78) season.

## Discussion

From what is already known about chacma baboon ecology, intra-population variation in ranging patterns of the baboons on SNR was expected. Suikerbosrand Nature Reserve is characterised by a heterogeneous landscape of habitat types within the reserve and is inhabited by about 687±77 SD baboons in at least 12 troops [43]. The data of the 10 troops analyzed in this study indicated that troop sizes ranged from 18–70 individuals with a mean troop size of 38 (±19.06 SD; N = 10). The mean troop size of baboon troops in the Blyde Canyon Nature Reserve, Mpumalanga, was determined to be 18.3 (± 6.8 SD; N = 21 troops) [46], whereas in forestry plantations along the Drakensberg escarpment in Mpumalanga the mean troop size was 43.1 (±11.1; N = 13 troops) [47]. The mean size of troops in the eastern parts of South Africa has been recorded to be 22.49 (N = 61) [48] whilst in the western part of South Africa troop sizes have been reported to range from 9 to 50 individuals [49, 50]. With the exception of one very large troop of 115 baboons, southern troops on the Cape Peninsula have a mean troop size of 34 (±16 SD; N = 12 troops) [24]. Although the population of baboons on SNR fall within reported troop sizes our results suggest that SNR baboon troop sizes resemble those of the Cape Peninsula and Forestry plantations.

The density of baboons on SNR is estimated to be between 3.57 and 4.06 ind/km^2^ which is higher than in Anderson’s [23] study on the same population, in which she estimated 3.2 ind/km^2^. Although SNR was declared a protected area in 1972, prior to this, baboons were considered vermin and persecuted indiscriminately by farmers in the general area. A 1974 census of the SNR baboons suggested a population of about 300 baboons and by Anderson’s 1981 study the population was estimated to be around 427 baboons. The increase in the population to around 687 as reported in the 2006 census [43] suggests that the baboons use the reserve as a protective refuge or that resources on the reserve are sufficient to maintain high numbers of baboons, suggesting that the population may have been, and possibly still is approaching its asymptote.

Although we suggest that troop sizes of the SNR population resemble those of the forestry plantations in the Mpumalanga Province of South Africa, the density of baboons on SNR was found to be higher than the Mpumalanga forestry plantation troops (2.8 ind/km^2^) [47] as well as Blyde Canyon Nature Reserve troops (1.8 ind/km^2^ ±0.4 SD) [46]. Suikerbosrand Nature Reserve baboon densities fall within that of baboons at Loskop dam in Mpumalanga of 3.7 ind/km^2^ [51] and the Cape Peninsula troops of 1.3 to 12.1 ind/km^2^ (mean 4.7±2.5 ind/ km^2^) [24]. In some populations such as those in the Drakensberg mountains, very low baboon densities occur (0.9–2.7 ind/km^2^: [4, 15, 48], whilst in the Kuiseb Canyon in Namibia and in the Okavango Delta in Botswana, a density of 5.3 ind/km^2^ and 24 ind/ km^2^ have been recorded respectively [13]. From the above comparisons, the SNR baboons fall well within the range of recorded baboon densities.

### Home range size and overlap

Despite the higher number of troops and density of baboons on SNR during our study, when compared to the study reported on by Anderson [23], who recorded mean home range sizes of 24.6 km^2^; our study found similar home range sizes of 26.1 km^2^±12.14 SD.

Based on published estimates, the home range sizes for chacma baboons in South Africa are suggested to be around 15.19 km^2^ [52], indicating that SNR troops have large home ranges. For example: Blyde Canyon Nature Reserve Mpumalanga baboons had a mean home range of 10.2 km^2^±2.3 SD [46], Mpumalanga forestry plantation troops had a mean home range of 14.55 km^2^ [47] and troops on the Cape Peninsula had a mean home range of 11.0 km^2^ ± 6.80 km^2^ [21]. Baboon troops in the Drakensburg were recorded to have home ranges of 23 km^2^ in high altitudes and 12 km^2^ in low altitude troops [4].

Variation in seasonal home range size for baboon troops on SNR (11.06 km^2^ to 46.22 km^2^ (mean: 21.57±3.55 SD) in the cold/dry season and from 11.27 km^2^ to 40.12 km^2^ (mean: 21.43±3.25 SD) in the hot/wet season was greater than reported for other troops (e.g. 1.9–3.5 km^2^ in the Limpopo province of South Africa [53]; 2.1–6.5 km^2^ in the Okavango Delta, Botswana [13], but was consistent in terms of variations across troops as reported in a study on the Cape Peninsula baboon population [24]. Home ranges have been reported to be smaller in dry seasons compared to wet seasons in South Africa’s Mpumalanga Province [47] and although there were no within troop differences in seasonal home range sizes in this study, there was a shift in spatial use which is suggested to be correlated to the availability of resources in different plant communities at different times of the year. This will however be discussed in a future paper.

Cape Peninsula troops were reported to have a mean percentage home range overlap of 7.3 ± 4.9%, with six of the nine troops investigated overlapping with all other troops [24]. In our study 29 of 45 possible troop home range overlaps occurred, with degree of overlap varying between 0.28 and 27.96% during the warm/wet season and between 0.20 and 30.01% during the cold/dry season; this is much lower than recorded for other studies. Five of the 19 intertroop comparisons in our study that had overlapping home ranges increased their percentage of overlap during the cold/dry season suggesting that when resources are less abundant (e.g. in poorer habitats or in the dry season), that home ranges are more likely to overlap between troops. Whether there are areas within the reserve that have sufficient resources throughout the year, requires further investigation

### Home range and troop size

Our findings indicate weak associations between troop size and home range size, suggesting that troop size in SNR may not significantly influence home range size. Our results for the SNR baboons is in contrast to other South African studies that found positive correlations between troop size and home range size [14, 24, 46]. Positive relationships between troop size and home range size would be more likely in areas of low resource availability, necessitating troops forage further afield to meet their nutritional demands as troop sizes increase. Based on this assumption, our results suggest that resources on SNR are sufficient for the baboon populations on SNR.

### Daily distances travelled

Primates use several strategies to cope with seasonal food scarcity [27, 54] and respond to reduced food availability by either reducing daily distances travelled and feeding on lower quality food items, or increasing daily distances travelled to search for high-quality food items [26, 28, 29]. Mean daily distances recorded in this study (from 1.64 km (±0.09 SE) to 2.91 km (±0.09 SE) for cold/dry season; 2.85 km (±0.08 SE) to 4.14 km (±0.09 SE) for warm/wet season) were similar to those found in other studies on chacma baboons in South Africa [14, 21, 23, 55]. However, when compared to the daily distances recorded for baboons in general, daily distances travelled by SNR baboons are generally shorter: chacma (8.0 km), yellow (4.2 km) (*Papio cynocephalus*), olive (6.4 km) (*Papio Anubis*) and hamadryas (13.2 km) (*Papio hamadryas*) [27]. Since daily distances travelled and the distribution of resources are suggested to be closely linked [5], short daily distances are indicative of highly concentrated food sources. Food resources for baboons on SNR appear to be sufficiently concentrated, allowing for shorter daily distances than in areas where resources are more scattered. Longer daily distances during the warm/wet season in this study compared to that of the cold/dry season could be indicative of seasonal photoperiod fluctuations. During the warm/wet season on SNR, baboons have an extra 2–3 daylight hours in which to forage and engage in other activities. Although there is no significant difference in home range size between seasons within troops, we suggest that the baboons take advantage of the extra daylight hours in the warm/wet season and forage for longer while moving longer distances within their home ranges. Whilst daily distances are longer in the warm/wet season than in the cold/dry season, seasonal variation in home range size could be attributed to the baboons foraging in a smaller area of their home range during the warm/wet season, albeit for longer periods of time due to increased daylight hours than during the cold/dry season. This explains the increased home range size but shorter daily distances travelled during the cold/dry season for some of the troops. The significantly longer daily travel distances during the cold/dry season compared to those in the warm/wet season found in the Blyde canyon baboons was suggested to be related to resources scattered over a wide area, forcing the baboons to move further afield during the time of the year when resources are patchy [17]. Similar findings were found for baboons in the Cape in terms of dry and wet season distances travelled [56].

Segal [18] suggested that baboons within SNR cope with reduced food availability and increased energy demands during the cold/dry season by minimising foraging effort (i.e. by searching less, decreasing distance travelled and feeding on a larger variety of food items), whereas during the warm/wet season, baboons increase foraging effort (i.e. searching and consuming more, increasing distances travelled and feeding more selectively). This supports the longer daily distances travelled during the warm/wet season recorded for this study, in that baboons can afford to be more selective due to the abundance of resources available to them during this season.

### Daily distances travelled and troop size

Similar to Anderson’s [23] study and assumptions based on existing baboon ecology [5, 6] troop size noticeably affected daily distance travelled, especially during the cold/dry season. So, although home range size was not majorly influenced by troop size, the distances that a troop travelled during the cold/dry season when resources are more dispersed and less available, were influenced by troop size.

### Ranging within and outside reserve

Only one of the 10 troops in our study spent more of their time outside of the reserve than inside. There was no significant relationship between home range size and tendency of a troop to leave the reserve. Troops that raid farmland are likely to use the reserve as a refuge, foraging on farmland as quickly as possible and then retreating to the reserve for other activities such as rest and socialising. This is a documented response for baboons in areas that are both dangerous and resource rich [57, 58]. In such areas, baboons forage intensely in patches that are resource rich and that are considered high risk areas (i.e. agricultural crops), whilst spending the rest of their time in low risk areas (i.e. reserves or naturally vegetated areas). However, just because a troop leaves the reserve does not necessarily mean that the troop raids farmland. Most of the land around the reserve is not cultivated farmlands, but rather fragments of natural vegetation in which the baboons are actually spending most of their ‘outside reserve’ time. In a previous study on the same population, Pahad [19] suggested that troops that were regarded as ‘occasional leavers’ (troops which left the reserve but spent less than 5% of their time outside the reserve) were most likely to leave the boundaries of the reserve and raid surrounding farmland. For this study, the troop that spent the majority of its time outside the reserve, utilised natural vegetation outside the reserve as its main foraging area and only raided surrounding farms rarely [19]. Even if troops are raiding farmland close to the reserve, they still spend most of their time inside the reserve. Furthermore, baboons are only likely to raid the farmlands at certain times of the year when mature crops or other high energy foods are available, and the energy benefits received from raiding outweighs the risks [19].

## Conclusion

The aim of this study was to determine the spatial utilization of baboon troops on SNR over a period of 12 months. Baboon ecology theory states that baboons exhibit intra-population variation in home range size and ranging patterns, which our study supports. Although our study population falls within reported ranges for troop sizes and densities and was consistent in terms of expected variation across troops, SNR baboons appear to have larger home range sizes when compared to average home range sizes of baboons in South Africa. Core range overlap between troops was more pronounced during the cold/dry season supporting the suggestion that when resources are limited, home ranges are more likely to overlap between troops. Troop overlap during the cold/dry season may be in response to patchy resource availability, but this requires further investigation. With the exception of the troop that spent most of its time outside SNR, troop size did not influence the range size of the troops in our study.

In summary we found that the home range size of chacma baboon troops on SNR were not significantly influenced by troop size, but that daily distances a troop travelled, especially within the cold/dry season, was significantly influenced by troop size. Having a spatial representation of baboon troop home ranges within the SNR provides a foundation for further studies into the quantification of available resources food, water and availability of sleeping sites within identified home ranges. Information generated from this study can be used to develop a formal baboon management plan by SNR conservation managers to assist them in reducing human-baboon conflict, it also provides the basis for further research on this population of baboons. We suggest that management consider incorporating the information on baboon home ranges generated from our study into future tourist infrastructure planning endeavors such as the placement of picnic sites, camping sites or lodges. By avoiding the placement of tourist facilities in areas of high baboon activity, management can assist in reducing potential human-baboon conflict. In addition, our study identifies the troops that incorporate areas outside of the reserve into their home ranges. This information can be used as baseline information when planning mitigation strategies for communicating with farmers and other land owners who are affected by raiding baboons. Policies on how to deal with human-baboon conflict, possible non-lethal deterrent methods and problem individual baboons need to be developed. As this reserve resembles an island and baboons are not readily able to migrate to populations outside of the reserve, monitoring numbers and resource utilisation is essential.

Suggestions for future research:

- Quantify the availability of the different plant communities on the reserve to the baboon troops and incorporate this information into the home ranges of the different troops
- Quantify the resources available to baboons in the various plant communities and determine the diet of baboons on Suikerbosrand Nature Reserve
- Determine and quantify the resources that baboons utilize outside of the reserve.
- As Suikerbosrand Nature Reserve is in essence an island surrounded by increasing human development, the closest free-living baboon populations need to be identified and assessed to determine whether natural dispersal between these populations can still potentially occur.
- Investigate the effectiveness of non-lethal deterrents such as bear bangers, virtual fences and so forth to reduce human-baboon conflict on and around the reserve.
